# Maternal pre-pregnancy body mass index and mental health problems in early adolescents from the 2004 Pelotas birth cohort

**DOI:** 10.1038/s41598-022-18032-y

**Published:** 2022-08-24

**Authors:** Gabriela Callo Quinte, Tiago N. Munhoz, Alicia Matijasevich, Ina S. Santos

**Affiliations:** 1 Postgraduate Program in Epidemiology, Federal University of Pelotas, Rua Marechal Deodoro, 1160 - 3° Piso, Bairro Centro, Caixa Postal 464, Pelotas, RS Cep: 96020-220 Brazil; 2 School of Psychology, Federal University of Pelotas, Pelotas, Brazil; 3 Departamento de Medicina Preventiva, Faculdade de Medicina FMUSP, Universidade de São Paulo, São Paulo, SP Brasil

**Keywords:** Psychiatric disorders, Obesity, Psychology

## Abstract

Possible mechanisms by which maternal pre-pregnancy body mass index (BMI) programs offspring mental disorders in late childhood are not fully clarified. To assess the association between maternal BMI and mental health problems at 11 years old, we used data from the 2004 Pelotas birth cohort which comprised 4231 newborns. Maternal pre-pregnancy BMI was analyzed as underweight (< 18.5), normal (18.5–24.9), overweight (25.0–29.9), and obesity (≥ 30.0). Mental health problems were assessed at the child’s age of 11 years by the Strengths and Difficulties Questionnaire, total difficulties score and subscale scores (emotional symptoms, conduct problems, hyperactivity/inattention, and peer relationship problems), dichotomized into normal/borderline and abnormal category. The associations between maternal pre-pregnancy BMI and mental health problems in the whole sample and stratified by sex were estimated using crude and adjusted linear and logistic regression. Both linear and logistic regression showed that pre-pregnancy weight was associated with mental problems in early adolescents. Maternal pre-pregnancy obesity was associated with increased odds of total difficulty score among the whole sample. Boys whose mothers were pre-pregnancy overweight had higher odds of conduct problems (OR = 1.56; 95% CI: 1.13, 1.95), when compared to children of normal pre-pregnancy weight mothers, even after adjustments. Maternal pre-pregnancy obesity was associated with mental health problems in early adolescents; specifically, pre-pregnancy overweight increased the risk for conduct problems in 11 years old boys.

## Introduction

Obesity is a worldwide public health problem, even among women of reproductive age^1^. In a Brazilian pooled cohort, more than 30% of women started pregnancy overweight or obese^2^. Several studies have observed associations between maternal obesity before and during pregnancy with other intrauterine risk factors and pregnancy complications, such as gestational diabetes and pre-eclampsia^3^. Infants born to obese women have higher rates of neural tube defects^4^ and an increased risk of obesity^5^ and asthma^6^ later in life.

Recent studies concerning long-term child mental health outcomes suggest pre-pregnancy obesity is associated with numerous neurodevelopmental and neuropsychiatric events^7,8^. Compared to children of normal-weight mothers, children whose mothers were obese were more likely to have behavioral or emotional problems^9^, developmental delay^9^, peer problems^10^, internalizing and externalizing problems^11^, attention deficit hyperactivity disorder (ADHD) or autism spectrum disorder (ASD)^8,9,12^. Also, severe obesity at pre-pregnancy was associated with worse problem-solving and poorer executive functioning in areas of attention, inhibitory control, and working memory in children^13^.

Nevertheless, the association between maternal pre-pregnancy obesity and child mental health is inconsistent across the studies^14,15^. Hence, this study aimed to assess the association between maternal pre-pregnancy BMI and children’s mental health at 11.

## Results

Information about pre-pregnancy BMI was available for 3875 mothers and SDQ scores data for 3563 participants. A total of 3385 cohort participants (80.0% of the original sample), comprising 1644 girls and 1741 boys, had complete information on maternal pre-pregnancy BMI on SDQ and were included in the analyses. Mothers of participants who kept in this study were generally 20–34 years old, more educated, more likely to live with a husband or partner, multiparous and nonsmokers during pregnancy, as compared to those who were not included (Supplementary Table 1).

The distribution of maternal pre-pregnancy BMI according to co-variables is presented in Table 1. Overall, before pregnancy, 2.3% (95% CI: 1.8–2.9) of the mothers were underweight, 54.7% (95% CI: 53.1–56.4) were normal weight, 29.0% (95% CI: 27.5–30.6) were overweight and 13.9% (95% CI: 12.8–15.1) were obese. The prevalence of obesity was higher among mothers aged ≥ 35 years, from the lowest socioeconomic strata, with a low level of formal education, and who lived without a husband or partner.

**Table 1:** Maternal pre-pregnancy body mass index (BMI), according to maternal and child characteristics.

	Total n = 3875 (%)	Underweight n = 96 (%)	Normal n = 2138 (%)	Overweight n = 1105 (%)	Obese n = 536 (%)	*P*-value*
Total		2.3 (1.8–2.9)	54.7 (53.1–56.4)	29.0 (27.5–30.6)	13.9 (12.8–15.1)	
***Maternal characteristics***						
**Age(years)**						< 0.001
≤ 19	18.6	5.4	68.4	20.5	5.7	
20–34	67.9	2.0	54.9	29.2	13.9	
≥ 35	13.5	0.8	38.0	36.3	25.0	
**Skin color**						0.001
White	73.6	2.6	56.0	28.8	12.6	
Black	19.5	1.6	51.5	29.8	17.1	
Other	7.1	3.7	57.2	21.8	17.3	
**Family monthly income (quintiles)**						0.007
1st(lowest) to 3rd	87.9	2.5	54.4	28.8	14.4	
4th to 5th(highest)	12.1	2.7	62.1	26.0	9.2	
**Education (years)**						< 0.001
0–4	12.8	1.9	47.2	31.3	19.5	
5–8	40.1	2.9	55.3	27.6	14.2	
≥ 9	47.1	2.3	57.5	28.5	11.7	
**Living with husband/partner**						0.002
Yes	84.2	3.3	60.7	25.7	10.3	
No	15.8	2.3	54.1	29.0	14.5	
**Smoking during pregnancy**						0.170
Yes	26.4	3.0	57.2	26.5	13.3	
No	73.6	2.3	54.5	29.2	14.0	
***Child characteristics***						
**Sex**						0.804
Girls	47.9	2.5	54.2	28.5	14.4	
Boys	52.1	2.5	55.7	28.5	13.3	
**Low birth weight**						< 0.001
Yes	8.9	5.0	62.1	23.0	9.9	
No	91.1	2.2	54.5	29.1	14.2	

*X^2^ test.

Table 2 shows the prevalence of mental health problems with 95% CI, according to maternal pre-pregnancy BMI. The prevalence of any difficulty was higher among children of pre-pregnancy underweight (21.8%) and obese mothers (17.2%) than among children of pre-pregnancy normal-weight mothers (13.1%). Prevalence of hyperactivity was also higher among children of pre-pregnancy underweight (19.2%) and obese mothers (17.4%) as compared to children of normal-weight mothers (16.9%).

**Table 2:** – Prevalence of mental health problems (with 95% confidence interval) according to maternal pre-pregnancy body mass index, among 11-year-old children.

Outcome—SDQ	Any difficulty			Emotional symptoms			Conduct problems			Hyperactivity/inattention			Peer relationship problems		
Total	All	Boys	Girls	All	Boys	Girls	All	Boys	Girls	All	Boys	Girls	All	Boys	Girls
n (%)	466 (13.8)	279 (16.0)	187 (11.4)	683 (20.2)	333 (19.1)	350 (21.3)	438 (12.9)	247 (14.2)	191 (11.6)	539 (15.9)	360 (20.7)	179 (10.9)	445 (13.2)	263 (15.1)	182 (11.1)
95% CI	12.6–15.0	14.4–17.8	9.9–13.0	18.8–21.5	17.3–21.0	19.4–23.3	11.8–14.1	12.6–15.9	10.2–13.3	14.7–17.2	18.8–22.6	9.4–12.4	12.0–14.3	13.5–16.9	9.6–12.7
**Underweight**															
n (%)	17 (21.8)	10 (24.4)	7 (18.9)	20 (25.6)	10 (24.4)	10 (27.0)	13 (16.7)	7 (17.1)	6 (16.2)	15 (19.2)	11 (26.8)	4 (10.8)	12 (15.4)	7 (17.1)	5 (13.5)
95%CI	14.0–32.4	13.5–39.9	9.1–34.9	17.1–36.5	13.5–39.9	15.1–43.6	9.8–26.7	8.3–31.9	7.3–31.9	11.9–29.6	15.4–42.5	4.0–25.8	8.9–25.2	8.2–31.9	5.6–28.9
**Normal**															
n (%)	243 (13.1)	141 (14.7)	102 (11.5)	364 (19.6)	189 (19.6)	175 (19.6)	218 (11.8)	119 (12.4)	99(11.1)	313 (16.9)	209 (21.7)	104 (11.7)	239 (12.9)	150 (15.6)	89 (10.0)
95% CI	11.7–14.7	12.6–17.0	9.5–13.7	17.9–21.5	17.3–22.3	17.2–22.4	10.4–13.3	10.4–14.6	9.2–13.4	15.3–18.7	19.2–24.4	9.7–14.0	11.4–14.5	13.4–18.0	8.2–12.1
**Overweight**															
n (%)	125 (12.7)	83 (16.3)	42 (8.8)	194 (19.7)	88 (17.3)	106 (22.3)	136 (13.8)	86 (16.9)	50 (10.5)	129 (13.1)	87 (17.1)	42 (8.8)	130 (13.2)	71 (13.9)	59 (12.4)
95% CI	10.8–15.0	13.4–19.8	6.6–11.8	17.3–22.3	14.3–20.9	18.8–26.3	11.8–16.1	13.9–20.5	8.0–13.6	11.1–15.4	14.1–20.7	6.6–11.8	11.2–15.5	11.2–17.3	9.7–15.7
**Obese**															
n (%)	81 (17.2)	45 (19.6)	36 (14.9)	105 (22.2)	46 (20.0)	59 (24.5)	71 (15.0)	35 (15.2)	36 (14.9)	82 (17.4)	53 (23.0)	39 (12.0)	64 (13.6)	35 (15.2)	29 (12.0)
95% CI	14.0–20.9	14.9–25.2	11.0–20.0	18.7–26.3	15.3–25.7	19.5–30.3	12.1–18.6	11.1–20.5	11.0–20.0	14.2–21.1	18.0–28.9	15.8–28.6	10.8–17.0	11.1–17.3	8.4–17.0
*P*-value*	0.015	0.130	0.043	0.362	0.555	0.268	0.120	0.101	0.249	0.037	0.099	0.401	0.913	0.847	0.495

*P-value: *X*^2^ test.

When stratified by sex, the proportion of girls of pre-pregnancy underweight and obese mothers who had any difficulty (18.9% and 14.9%, respectively) and conduct problems (16.2% and 14.9%, respectively) was higher than among girls of pre-pregnancy normal weight mothers (11.5% and 11.1%, respectively). Boys from pre-pregnancy underweight and obese mothers had higher proportions of hyperactivity (26.8% and 23.0%, respectively) when compared to boys from pre-pregnancy normal-weight mothers (21.7%) (Table 2).

In unadjusted linear analyses, there was a significant increase of 0.05 SDs in peer relationship problems with every SD increase in pre-pregnancy weight (*p* = 0.03). (Table 3). The logistic regression analysis orientated the results to other findings. Children of pre-pregnancy obese mothers had increased odds of having any difficulty (OR = 1.38, 95% CI: 1.05–1.81) and conduct problems (OR = 1.33, 95% CI: 1.00–1.78) compared with children of pre-pregnancy normal weight mothers. Boys of pre-pregnancy overweight mothers had increased odds of conduct problems (OR = 1.44, 95% CI: 1.07–1.95) compared with their counterparts of pre-pregnancy normal weight mothers. On the other hand, we did not find any association between girls of pre-pregnancy obese mothers and any mental health problems. (Table 4).

**Table 3:** Linear regression associations between maternal pre-pregnancy body mass index (BMI) and children’s mental problems at 11 years of age.

Outcome—SDQ	Any difficulty (βs) (*p*-value)		Emotional symptoms (βs) (*p*-value)		Conduct problems (βs) (*p*-value)		Hyperactivity/ inattention (βs) (*p*-value)		Peer relationship problems (βs) (*p*-value)	
	Crude	Adjusted	Crude	Adjusted	Crude	Adjusted	Crude	Adjusted	Crude	Adjusted
**Maternal pre-pregnancy BMI**										
All	0.008 (0.649)	0.02 (< 0.001)	0.02 (0.370)	0.02 (< 0.001)	0.23 (0.179)	0.04 (< 0.001)	−0.03 (0.123)	0.01 (< 0.001)	0.03 (0.086)	0.04 (< 0.001)
Boys	−0.01 (0.836)	0.01 (< 0.001)	0.01 (0.779)	0.01 (0.144)	0.02 (0.334)	0.03 (< 0.001)	−0.03 (0.150)	−0.02 (< 0.001)	0.01 (0.757)	0.02 (< 0.001)
Girls	0.03 (0.288)	0.05 (< 0.001)	0.02 (0.353)	0.03 (< 0.001)	0.03 (0.297)	0.05 (< 0.001)	−0.01 (0.697)	0.02 (0.536)	0.05 (0.03)	0.10 (< 0.001)

^a^Adjusted for child’s sex, maternal age, skin color, parity, maternal schooling, family income and smoking during pregnancy (child´s sex was removed when appropriated).

*p*-value: Prob > F.

**Table 4:** Logistic regression associations between maternal pre-pregnancy body mass index (BMI) and children’s mental problems at 11 years of age.

Outcome—SDQ	Any difficulty		Emotional symptoms		Conduct problems		Hyperactivity/ inattention		Peer relationship problems	
	OR (95% CI)		OR (95% CI)		OR (95% CI)		OR (95% CI)		OR (95% CI)	
Maternal pre-pregnancy BMI	Crude	Adjusted	Crude	Adjusted	Crude	Adjusted	Crude	Adjusted	Crude	Adjusted
All	*p* = 0.016	*p* = 0.034	*p* = 0.363	*p* = 0.492	*p* = 0.121	*p* = 0.050	*p* = 0.038	*p* = 0.121	*p* < 0.912	*p* = 0.953
< 18.5	1.85 (1.1, 3.21)	1.72 (0.97, 3.04)	1.41 (0.84, 2.37)	1.38 (0.82, 2.34)	1.50 (0.81, 2.77)	1.37 (0.73, 2.58)	1.17 (0.66, 2.08)	1.09 (0.60, 1.97)	1.23 (0.65, 2.31)	1.14 (0.60, 2.16)
18.5–24.9	1.00	1.00	1.00	1.00	1.00	1.00	1.00	1.00	1.00	1.00
25.0–29.9	0.97 (0.77, 1.22)	1.04 (0.81, 1.32)	1.01 (0.83, 1.22)	1.01 (0.82, 1.23)	1.20 (0.96, 1.52)	1.32 (1.03, 1.68)	0.74 (0.60, 0.93)	0.79 (0.63, 0.99)	1.03 (0.82, 1.29)	1.05 (0.83, 1.34)
≥ 30	1.38 (1.05, 1.81)	1.42 (1.06, 1.91)	1.17 (0.92, 1.50)	1.14 (0.88, 1.47)	1.33 (1.00, 1.78)	1.42 (1.04, 1.93)	1.04 (0.79, 1.36)	1.09 (0.82, 1.45)	1.0 (0.79, 1.43)	1.05 (0.77, 1.44)
**Boys**	*p* = 0.134	*p* = 0.201	*p* = 0.556	*p* = 0.590	*p* = 0.102	*p* = 0.055	*p* = 0.101	*p* = 0.205	*p* = 0.847	*p* = 0.841
< 18.5	1.88 (0.90, 3.92)	1.73 (0.81, 3.67)	1.32 (0.64, 2.74)	1.30 (0.62, 2.71)	1.46 (0.63, 3.36)	1.32 (0.56, 3.12)	1.32 (0.65, 2.68)	1.22 (0.59, 2.52)	1.11 (0.49, 2.56)	1.08 (0.46, 2.51)
18.5–24.9	1.00	1.00	1.00	1.00	1.00	1.00	1.00	1.00	1.00	1.00
25.0–29.9	1.14 (0.85, 1.53)	1.18 (0.87, 1.61)	0.86 (0.65, 1.13)	0.85 (0.64, 1.13)	1.44 (1.07, 1.95)	1.56 (1.13, 2.16)	0.74 (0.56, 0.98)	0.77 (0.58, 1.03)	0.88 (0.65, 1.19)	0.87 (0.63, 1.19)
≥ 30	1.42 (0.98, 2.05)	1.41 (0.95, 2.09)	1.02 (0.71, 1.47)	0.98 (0.67, 1.42)	1.27 (0.85, 1.91)	1.33 (0.85, 2.06)	1.08 (0.77, 1.52)	1.09 (0.76, 1.57)	0.97 (0.65, 1.45)	0.97 (0.64, 1.48)
**Girls**	*p* = 0.046	*p* = 0.080	*p* = 270	*p* = 0.234	*p* = 0.252	*p* = 0.250	*p* = 0.404	*p* = 0.589	*p* = 0.339	*p* = 0.448
< 18.5	1.80 (0.77, 4.22)	1.70 (0.71, 4.07)	1.52 (0.72, 3.19)	1.43 (0.67, 3.04)	1.55 (0.63, 3.80)	1.48 (0.59, 3.72)	0.92 (0.32, 2.64)	0.85 (0.29, 2.48)	1.02 (0.42, 2.49)	1.41 (0.52, 3.80)
18.5–24.9	1.00	1.00	1.00	1.00	1.00	1.00	1.00	1.00	1.00	1.00
25.0–29.9	0.75 (0.51, 1.09)	0.82 (0.56, 1.22)	1.18 (0.90, 1.54)	1.21 (0.91, 1.61)	0.94 (0.66, 1.35)	1.03 (0.71, 1.49)	0.73 (0.50, 1.07)	0.81 (0.55, 1.19)	1.27 (0.95, 1.69)	1.33 (0.92, 1.93)
≥ 30	1.36 (0.90, 2.05)	1.46 (0.94, 2.28)	1.33 (0.95, 1.86)	1.38 (0.97, 1.97)	1.40 (0.93, 2.12)	1.51 (0.97, 2.34)	1.04 (0.67, 1.61)	1.12 (0.70, 1.79)	1.28 (0.89, 1.84)	1.21 (0.75, 1.95)

^a^Adjusted for child’s sex, maternal age, skin color, parity, maternal schooling, family income and smoking during pregnancy (child´s sex was removed when appropriated).

*p*-value: post estimation test.

The strength of the associations of pre-pregnancy maternal overweight and obesity with mental health problems increased after adjusting for confounders for linear regression. For logistic regression, the association increased specifically for conduct problems.

There was a significant increase of 0.02, 0.04 and 0.04 SDs in emotional symptoms, conduct problems and peer relationships problems with every SD increase in pre-pregnancy weight, respectively (*p* < 0.01). In boys, the increase was 0.03 SDs in conduct problems score and 0.02 SDs in peer relationship problems with every SD increase in pre-pregnancy weight, respectively (*p* < 0.01). In contrast, there was a decrease of -0.02 SDs in hyperactivity score with every SD increase in pre-pregnancy weight (*p* < 0.01) (Table 3). On the other hand, the associations of pre-pregnancy maternal obesity with any difficulty (OR = 1.42, 95% CI: 1.06–1.91) and conduct problems (OR = 1.42, 95%CI: 1.04–1.93) increased after adjusting for confounders, even among boys whose mothers were overweight in pre-pregnancy (OR = 1.56, 95% CI: 1.13–2.16) (Table 4).

## Discussion

In this study, maternal pre-pregnancy overweight and obesity were associated with having any difficulty among children at eleven years of age. Maternal pre-pregnancy weight was found associated with emotional problems, conduct problems, peer relationship problems, hyperactivity/inattention problems among children at eleven years. Regardless of the type of analysis, boys of pre-pregnancy overweight mothers had higher odds of presenting conduct problems even after adjustment for confounders compared to children of normal pre-pregnancy weight mothers.

Our findings are in concordance with previously published studies. Kong et al. found that maternal pre-pregnancy obesity was associated with an increased risk of offspring’s psychiatric and mild neurodevelopmental disorders, primarily conduct disorders^16^. Similar results were demonstrated in the study by Menting et al. carried out on children from Amsterdam^17^. In this study, the authors assessed child behavioral problems with the maternal and teacher version of the SDQ, finding that maternal pre-pregnancy overweight and obesity were associated with increased children's behavior problems. Teachers reported peer relationship problems for children whose mothers were obese before pregnancy^17^. Another study of 1311 mother–child pairs reported that children of obese class II/III mothers had increased odds of emotional symptoms, peer relationship problems, total psychosocial difficulties, ADHD diagnosis, autism, or developmental delay diagnosis. In addition, they were more likely to need speech-language therapy, psychological services, and any special needs service compared with children of normal weight mothers^10^. Others have reported associations between maternal pre-pregnancy obesity and lower cognitive performance^18^, intellectual disability^19,20^, ADHD^21^, and autism^22^. Although the specific outcomes differed, the findings are generally consistent with those observed in our study. Only one study, based on two European pregnancy cohorts-ALSPAC (UK) and Generation R (Netherlands) found no evidence between maternal pre-pregnancy overweight and offspring cognitive development and behavioral problems^14^.

Some mechanisms to explain the association between maternal obesity and offspring mental disorders have been explored. Using sibling comparisons, Chen et al. studied high maternal pre-pregnancy body mass index associated with increased risk of offspring Attention-deficit/hyperactivity disorder. They observed the associations between pre-pregnancy overweight/obese mothers with an increased risk of ADHD no longer remained when complete sibling comparisons were performed. Their results suggested that the association between maternal pre-pregnancy overweight/obesity and offspring ADHD could be ascribed to unmeasured familial confounding, which might reflect genetic origin.

On the other hand, the association of pre-pregnancy overweight and obesity with maternal and neonatal adverse outcomes could lead to changes in altered maternal and neonatal physiology, as reported in the meta-analysis by Vats and colleagues^23^. They included 86 cohort studies of pregnant women and found that pre-pregnancy overweight and obese mothers were associated with cesarean delivery, emergency cesarean delivery, gestational diabetes, gestational hypertension, labor induction, postpartum hemorrhage, pre-eclampsia and pre-term premature rupture of membrane. The fetus and neonates of overweight and obese mothers have a higher risk for admission to the newborn intensive care unit, low APGAR score, large for gestational age, macrosomia, preterm birth. These adverse outcomes directly contribute to malprogramming^23^.

Furthermore, altered maternal physiology may result in increased inflammation^24^, lipotoxicity^25^, and oxidative stress in the fetoplacental unit. These changes in the *in-utero* environment may contribute to the malprogramming of the fetal brain, pancreas, skeletal muscle, and adipose tissue, among other organs. Peripheral inflammation and insulin resistance lead to central insulin resistance and aberrant central glucose metabolism and transport. Moreover, elevated leptin levels and leptin resistance are prominent in obese mothers^26^. Considered a critical neurotrophic factor, leptin may signal abnormalities during fetal development, such as decreased neuronal stem cell differentiation and growth^27^, and the regulation of synaptic plasticity and neurotransmitter receptor trafficking^28^. Thus, during crucial developmental periods, leptin signaling is considered a potential mechanism underlying abnormal neurodevelopment in fetuses of obese women^29^. Thus, resulting in an increased risk for offspring mental health problems^12^.

### Strengths and limitations

Our study has some strengths and limitations. The large population-based sample, face-to-face interviews with adolescents and their caregivers, and the use of an internationally recognized instrument to screen mental health problems are among the study’s strengths. Being validated in Brazil and applied by trained psychologists resulted in high-quality data acquisition. On the other hand, the SDQ was applied only to the mothers/caregivers. It would have been interesting to use the SDQ to the teachers and the children’s pairs, too, to obtain a more accurate profile of the cohort participant’s mental health. Another limitation is the presence of attrition in our sample, which could potentially lead to selection bias. Nonetheless, the sample loss was at-random, and the follow-up rates were higher among non-LBW children, from highly educated mothers and non-smokers mothers during pregnancy. These characteristics are commonly associated with adequate child mental health^30,31^. Therefore, it is unlikely that the observed associations were due to attrition bias.

Our findings evidence associations of maternal pre-pregnancy obesity with children’s mental health problems at eleven years of age. Specifically, boys of pre-pregnancy obese mothers had a higher risk of developing conduct problems when compared with children of normal pre-pregnancy weight mothers. This study highlights that pre-pregnancy obesity may have long-term mental health effects on early adolescents, supporting previous evidence that recommends preventive preconception health care.

## Methods

The 2004 Pelotas birth cohort is a population-based birth cohort of individuals born to women living in the urban area of Pelotas, a southern city of Brazil, from January 1st to December 31st, 2004. A total of 4231 live births from all five city hospitals visited daily were enrolled in the study (99.2% of the eligible population). Trained personnel conducted structured interviews containing questions about the demographic, socioeconomic, behavioral, and biological characteristics of mothers and children at different stages. These included the perinatal study (within the first 24 h after delivery); at 3, 12, 24, and 48 months; and at 6 and 11 years of age. Detailed method information can be found elsewhere^32,33^.

For this study, we used information collected at birth and the 11-year follow-up. In the perinatal study, the interviews and newborn examinations were carried out in maternity hospitals. The response rate of the 11-year follow-up was 87%. All evaluations of this follow-up were performed at the research clinic of the Postgraduate Program in Epidemiology of the Federal University of Pelotas and, we used RedCap as the instrument for data collection^34^.

### Maternal pre-pregnancy BMI

The primary exposure was maternal pre-pregnancy BMI. To construct this variable, the mother pre-pregnancy weight was collected via antenatal record card (n = 3270) when available or by the maternal report (n = 3911) during the perinatal study. Height was collected during the 3-month follow-up visit by our trained field team using an aluminum stadiometer with 1 mm precision (n = 3974). Pre-pregnancy BMI was calculated in kg/m^2^ and categorized according to the World Health Organization standards. These categories were underweight (BMI < 18.5), normal weight (BMI 18.5–24.9), overweight (BMI 25.0–29.9), and obesity (BMI ≥ 30.0)^35^.

### Children mental health assessment

At the 11-year follow-up, trained psychologists interviewed participants’ mothers or caregivers using the Strengths and Difficulties Questionnaire (SDQ), a brief screening instrument designed to assess mental health problems in children and adolescents in the last six months. Developed by Goodman R., the SDQ was validated by Fleitlich-Bilyk and colleagues for use in Brazilian population^36,37^.

The instrument was composed of 25 questions divided into five subscales: emotional symptoms, conduct problems, hyperactivity/inattention, peer relationship problems, and prosocial behavior. Each question was scored on a three-point scale (not true = 0; somewhat true = 1; certainly true = 2). The total difficulty score (any difficulty), calculated by adding the results of the subscales (except prosocial behavior), ranged from 0 to 40. The subscale scores ranged from 0 to 10. We used the total SDQ score and the four subscales (emotional symptoms, conduct problems, hyperactivity/inattention, and peer relationship problems) dichotomized into normal/borderline and abnormal categories. We adopted the suggested cutoff points for parents’ responses available on the SDQ website (https://sdqinfo.org/py/sdqinfo/c0.py): ≥ 14 for any difficulty, ≥ 4 for emotional symptoms, ≥ 3 for conduct problems, ≥ 6 for hyperactivity/inattention, and ≥ 3 for peer relationship problems.

### Co-variables

The variables considered potential confounders were collected at the perinatal study and chosen a priori*,* based on previous research exploring associations between maternal weight and child development. From the mother, we used the following items: family monthly income (in Brazilian Real), later analyzed in quintiles (1st quintile comprised the poorest and 5th the wealthiest group); maternal schooling (0–4, 5–8 and ≥ 9 years of formal education); mother´s age (≤ 19, 20–34 and ≥ 35 years); the presence of a husband or partner (yes, no); self-referred skin color (White, Black, Other); parity (1, 2 and ≥ 3); smoking during pregnancy-at least one cigarette per day, every day, during any trimester of pregnancy (yes, no); alcohol use during pregnancy-regular use at least once a week, regardless of intake amount (yes, no).

From the child, we used low birth weight (LBW) (< 2500 g; yes, no) to describe the children included in this study, and sex (male, female) to test effect modification.

### Analysis

The sample distribution and the prevalence of maternal pre-pregnancy BMI categories were analyzed according to maternal and child characteristics. We used the continuous SDQ scores and also compared the proportion of children with total difficulties and each SDQ subscale score (emotional symptoms, conduct problems, hyperactivity/inattention, and peer relationship problems) according to maternal pre-pregnancy BMI by using bivariate analyses and stratifying by sex. We used linear regression to obtain standarised coefficients (βs) indicating the change on the SDQ subcategories score by a change in the mother’s weight. Also, we used logistic regression to obtain odds ratios (OR) with corresponding 95% confidence intervals (95% CI) for the association between maternal pre-pregnancy BMI and child mental health outcomes. A *p*-value < 0.05 was considered to be statistically significant. All the crude and adjusted analyses were conducted for the sample as a whole. Interaction terms were tested to examine whether the association between maternal pre-pregnancy BMI and child mental health differed by sex. Due to finding interaction between maternal pre-pregnancy BMI and sex (overweight in girls *p* = 0.003 and overweight in boys *p* = 0.024), we stratified the analysis by sex. We performed all analyses using Stata program, version 14.0 (StataCorp LP, College Station, TX).

### Ethics

The study was approved by the Ethics and Research Committee of the Faculty of Medicine of the Federal University of Pelotas and adhered to the ethical guidelines of clinical and epidemiological research. Mothers or legal guardians of the children signed an Informed Consent Form after having been notified and explained about the study. At the 11-year follow-up, the children also signed an Informed Consent Form.

## Supplementary Information

Supplementary Information.

## Data Availability

The datasets used and analyzed during the current study are available from the corresponding author on reasonable request.
