# Supplementary material for: Maternal pre-pregnancy body mass index and mental health problems in early adolescents from the 2004 Pelotas birth cohort
Source: Sci Rep. 2022 Aug 24;12:14437. doi: 10.1038/s41598-022-18032-y (PMC9402554; doi:10.1038/s41598-022-18032-y)
Supplement: Supplementary file 1 — Supplementary Information. [file 41598_2022_18032_MOESM1_ESM.docx]

Supplementary table 1. Baseline characteristics of mothers and infants included and not included in the study.

|  |  | **Included**  **n=2518** | **Not Included**  **n=1713** | **P-value** |
| --- | --- | --- | --- | --- |
|  |  |  |  |  |
| ***Maternal characteristics*** | |  |  |  |
| **Age(years)** |  |  |  | 0.022 |
| ≤19 |  | 17.7 | 20.8 |  |
| 20-34 |  | 68.4 | 66.9 |  |
| ≥35 |  | 14.0 | 12.3 |  |
| **Skin color** |  |  |  | 0.123 |
| White |  | 74.1 | 56.0 |  |
| Black |  | 19.0 | 51.5 |  |
| Other |  | 7.0 | 57.2 |  |
| **Family monthly income (quintiles)** | |  |  | 0.032 |
| 1^st^(lowest) to 3^rd^ |  | 88.5 | 90.6 |  |
| 4^th^ to 5^th^(highest) |  | 11.5 | 9.4 |  |
| **Education (years)** |  |  |  | <0.001 |
| 0-4 |  | 12.1 | 20.3 |  |
| 5-8 |  | 40.4 | 42.4 |  |
| ≥ 9 |  | 47.5 | 36.5 |  |
| **Living with husband/partner** | | |  | <0.001 |
| Yes |  | 85.3 | 81.1 |  |
| No |  | 16.7 | 18.9 |  |
| **Smoking during pregnancy** | |  |  | 0.0.12 |
| Yes |  | 26.1 | 29.6 |  |
| No |  | 73.9 | 70.4 |  |
| ***Child characteristics*** | |  |  |  |
| **Sex** |  |  |  | 0.984 |
| Girls |  | 48.1 | 48.1 |  |
| Boys |  | 51.9 | 51.9 |  |
| **Low birth weight** |  |  |  | 0.002 |
| Yes |  | 8.9 | 11.7 |  |
| No |  | 91.1 | 88.3 |  |
